# Common and specific genetic basis of metabolite-mediated drought responses in rice

**DOI:** 10.1007/s44154-024-00150-4

**Published:** 2024-01-23

**Authors:** Zilong Guo, Shouchuang Wang, Feng Zhang, Denghao Xiang, Jun Yang, Dong Li, Baowei Bai, Mingqiu Dai, Jie Luo, Lizhong Xiong

**Affiliations:** 1 National Key Laboratory of Crop Genetic Improvement, Hubei Hongshan Laboratory, Huazhong Agricultural University, Wuhan, 430070 China; 2 Fujian Agriculture and Forestry University, Fuzhou, 350002 China; 3 Sanya Nanfan Research Institute of Hainan University, Hainan Yazhou Bay Seed Laboratory, Sanya, 572025 China

**Keywords:** Drought-responsive metabolite, Natural variation, Drought response, Comparative GWAS, Rice

## Abstract

Plants orchestrate drought responses at metabolic level but the genetic basis remains elusive in rice. In this study, 233 drought-responsive metabolites (DRMs) were quantified in a large rice population comprised of 510 diverse accessions at the reproductive stage. Large metabolic variations in drought responses were detected, and little correlation of metabolic levels between drought and normal conditions were observed. Interestingly, most of these DRMs could predict drought resistance in high accuracy. Genome-wide association study revealed 2522 significant association signals for 233 DRMs, and 98% (2471/2522) of the signals were co-localized with the association loci for drought-related phenotypic traits in the same population or the linkage-mapped QTLs for drought resistance in other populations. Totally, 10 candidate genes were efficiently identified for nine DRMs, seven of which harbored *cis*-eQTLs under drought condition. Based on comparative GWAS of common DRMs in rice and maize, representing irrigated and upland crops, we have identified three pairs of homologous genes associated with three DRMs between the two crops. Among the homologous genes, a transferase gene responsible for metabolic variation of *N*-feruloylputrescine was confirmed to confer enhanced drought resistance in rice. Our study provides not only genetic architecture of metabolic responses to drought stress in rice but also metabolic data resources to reveal the common and specific metabolite-mediated drought responses in different crops.

**Supplementary Information:**

The online version contains supplementary material available at 10.1007/s44154-024-00150-4.

## Introduction

To avoid global food shortage by 2050, it is estimated that an annual increase of 44 million metric tons in food production is urgently needed (Tester and Langridge 2010). Scarce and unpredictable water resources usually cause drought stress in most rain-fed areas of Asia and Africa (Rockstrom et al. 2007), which severely threatens crop production and food security. Rice (*Oryza sativa* L.) feeds more than half of the world’s population while its yield and quality are highly vulnerable to drought stress (Dien et al. 2019). Genetic improvement of drought resistance (DR) is the most important approach to reduce yield loss due to drought, and many efforts have been made in genetic mapping and validation of QTLs in rice (Hu and Xiong 2014). However, DR is a complex trait that is governed by a number of minor-effect genes with diverse molecular mechanisms, which results in insufficient knowledge of its genetic bases (Fukao and Xiong 2013). Besides, most phenotypic traits used for DR evaluation are not adequately effective due to low heritability (such as grain yield), subjectivity (such as leaf drying and rolling scores), or bias caused by growth and development heterogeneity (such as varying plant size and flowering time) and environment heterogeneity (especially for soil moisture status) (Hu and Xiong 2014). To address the dilemma of DR evaluation, several efforts involving optics-based phenotyping have been made, and highly heritable image-traits have been invented to dynamically monitor drought responses and evaluate DR under the pot-grown and the actual field conditions (Guo et al. 2018; Jiang et al. 2021). Although many DR-related QTLs have been reported, the large interval of QTL and the complex molecular mechanisms involved in DR preclude the rapid identification of candidate genes (Blum 2011). Evaluating DR is bias-prone and labor-intensive and a gene’s effect could be masked by environmental noise, especially for a large mapping population. Fortunately, construction and phenotyping of a large secondary population is not the only approach for QTL fine-mapping and cloning in the omics era (Broekema et al. 2020). Metabolites can bridge the gap between DNA sequence variation and complex traits (Langridge and Fleury 2011), and thus have potentials to reveal genetic architecture of DR and provide valuable clues for causal genes.

Understanding plant metabolic responses to drought stress benefits the dissection of DR (Obata and Fernie 2012; Zhang et al. 2022). Previous studies show that a number of metabolites collectively response to drought and they function in antioxidant and osmotic adjustment (Fabregas and Fernie 2019; Nakabayashi and Saito 2015). Abscisic acid (ABA) is well-known for its functions in drought response, and other phytohormones, such as cytokinins (CK), also collectively cope with drought stress (Ullah et al. 2018). As primary metabolites, sugars (such as glucose and fructose) and amino acids (such as proline and tryptophan) are induced to protect plants from drought stress (Fabregas and Fernie 2019). As the largest group of plant-specialized metabolites, polyphenols (including phenolic acids, flavonoids, stilbenoids, and lignans), are involved in stress resistance in plants (Samec et al. 2021). Polyamines are essential compounds in most living organisms and participate in drought response and resistance dependent on ABA. Drought-responsive genes confer enhanced DR by altering the levels of metabolites involving endogenous ABA, putrescine, proline, acetate, trehalose, and flavonoids (Minocha et al. 2014). Metabolome-based genome-wide association study (mGWAS) is an efficient approach to identify metabolic genes, but most of the mGWAS experiments were performed under well-water condition (Chen et al. 2014; Fang and Luo 2019; Wen et al. 2014). Recently, mGWAS under drought condition was performed in maize (an upland crop) and two metabolic genes conferring enhanced DR were discovered (Zhang et al. 2021). However, natural metabolic variation under drought condition in rice (a lowland or irrigated crop that is vulnerable to drought) and comparative mGWAS under drought condition in different crops has not been addressed.

Our previous study proposed a novel integrated method of detecting, identifying, and quantifying widely targeted metabolites and studied metabolic responses to drought stress of two rice varieties – IRAT109 and ZS97 (Chen et al. 2013). We utilized an optics-based phenotyping facility in another previous work to study the genetic architecture of drought responses using a natural population consisting of 510 rice germplasms (Guo et al. 2018). To fully understand the metabolic variations under drought condition, the metabolites were simultaneously quantified in the same population when subjected to the optics-based drought phenotyping, and the mGWAS results were integrated with the phenome-based GWAS results to further dissect the genetic basis of drought responses in rice.

In this study, hundreds of drought-responsive metabolites were identified and these metabolites could predict DR in high confidence. Candidate genes of mGWAS loci were efficiently explored for diverse DRMs in rice. Furthermore, we compared the metabolite-mediated drought responses and their genetic bases in rice and maize and found that common and specific metabolite-mediated drought responses co-exist and are underlain by both homologous and non-homologous genes in the two representative crops.

## Results

### Metabolic profiling of drought-responsive metabolites in rice under drought condition

**Fig. 1:**
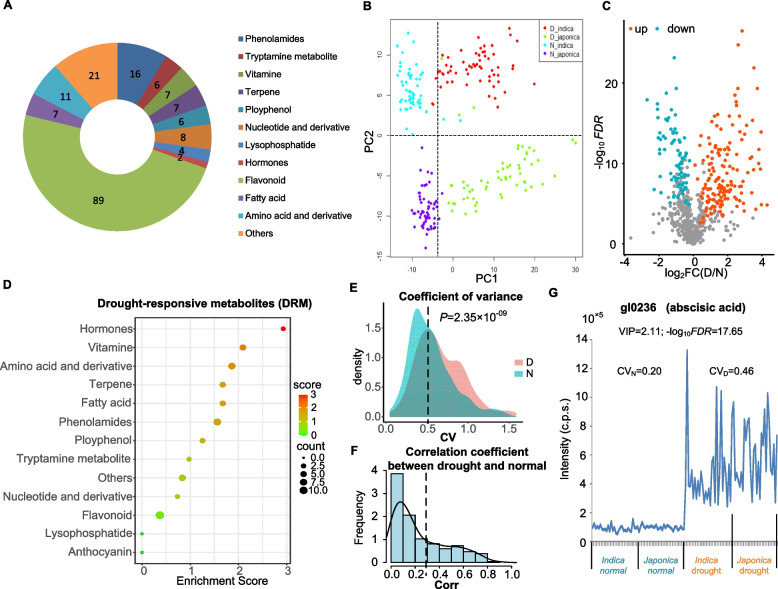
Characterization of the drought-responsive metabolites. **A** Groups of 184 annotated metabolites. **B** PCA of 60 rice accessions (including 30 *indica* and 30 *japonica* accessions) under drought and normal conditions based on the 682 distinct metabolic features. Red, green, blue, purple dots represent *indica* accessions under drought condition, *japonica* accessions under drought condition, *indica* accessions under normal condition, *japonica* accessions under normal condition, respectively. **C** Volcano plot of drought-responsive metabolites (DRMs). Orange and cyan dots represent up-regulated and down-regulated DRMs, respectively. FDR values were calculated based on paired *t*-test. **D** Groups of DRMs and enrichment analyses. **E** Distribution of coefficients of variance (CV) of DRMs under drought and normal conditions, respectively. **F** Distribution of correlation coefficients of DRMs’ levels between the two water conditions (drought and normal conditions) **G** Intensity of abscisic acid (ABA) under drought and normal conditions

To identify drought-responsive metabolites (DRMs) in rice, leaf metabolic samples from 60 drought-sensitive or tolerant rice varieties (including 30 *indica* and 30 *japonica*) from a core germplasm panel were collected for metabolic profiling under drought stress and normal irrigation conditions. By liquid chromatography–mass spectrometry, 682 distinct metabolite features, including 184 annotated metabolites, were successfully detected and quantified (Supplementary Table 1). The 184 known metabolites were assigned into 11 major groups (hormone, tryptamine metabolites, phenolamide, polyphenol, amino acid and derivative, nucleotide and derivative, flavonoid, terpene, lysophosphatide, vitamin, and fatty acid) (Fig. 1A). Based on principal component analysis (PCA) for the 682 metabolic features of the 60 accessions under two water regime conditions (Supplementary Table 2), the first principal component (PC1) obviously discriminates the accessions under two treatments, indicating that drought stress predominantly disturbs rice metabolome and the metabolic changes are related to the rice responses to drought stress (Fig. 1B), and the second principal component (PC2) discriminates *indica* and *japonica* accessions (Fig. 1B). Further, we found that very few metabolites were overlapped between drought-normal differentiated metabolic features and *indica-japonica* differentiated features, suggesting metabolic responses to drought stress may not be associated with *indica-japonica* differentiation.

A metabolic feature with VIP (variable importance for the projection)≧1 and *FDR* (false discovery rate) of paired *t*-test < 0.01 was defined as DRMs, and a total of 233 DRMs were determined (Fig. 1C and Supplementary Table 3). Enrichment analysis showed that these DRMs were annotated to a variety of different metabolic pathways such as hormones and phenolamides (Fig. 1D), suggesting the complexity of metabolite-mediated drought responses. Based on the DRMs, the accuracy rate in discriminating the accessions under the two water conditions was 99.17% using linear discriminant analysis. Coefficients of variance (CV) of DRM levels under drought condition were significantly greater than those under normal condition (*P* = 2.35 × 10^−9^, paired *t*-test; Fig. 1E and Supplementary Table 3). For 69% (160/233) of the DRMs, metabolic levels under drought condition showed weak correlation to those under normal condition (absolute Pearson correlation coefficients |R| < 0.3) (Fig. 1F; Supplementary Table 3). ABA, known as a abiotic stress hormone (Nambara and Marion-Poll 2005), was identified as a typical DRM in the hormone group (VIP = 2.11, −log_10_*FDR* = 17.65), and it showed elevated level on average at population scale and large variation under drought condition (fold change of drought / normal (FC) =7.74; CV__D_ = 0.46, CV__N_ = 0.20), and Pearson correlation coefficient (PCC) between the two water conditions was only 0.08 (*P* = 0.42) (Fig. 1G), indicating large variation of drought responses at metabolic level. Besides, many new DRMs were identified in this study. For example, flavonoids function as antioxidants to scavenge ROS caused by abiotic stress, such as UV-B radiation (Fini et al. 2011), but the role of flavonoids in drought response has not been well-characterized. Out of 87 flavonoids in the study, 11 flavonoids belonging to DRMs were down-regulated under drought condition, of which *C*-hexosyl-luteolin *O*-hexoside showed dramatic reduction (FC = 0.23, VIP = 1.83) and larger metabolic variation under drought condition than that under normal condition (CV__D_ = 1.08, CV__N_ = 0.58). These results imply a necessity to decode the natural metabolic variations under drought condition.

### Metabolite-based prediction of drought resistance in rice

To test whether DRMs can predict DR, we quantified the DRMs under drought condition in a large core collection comprised of 510 diverse rice accessions (Supplementary Table 4), which were simultaneously phenotyped by both non-destructive optical phenotyping facility and manual measurements in our previous study (Guo et al. 2018).

**Fig. 2:**
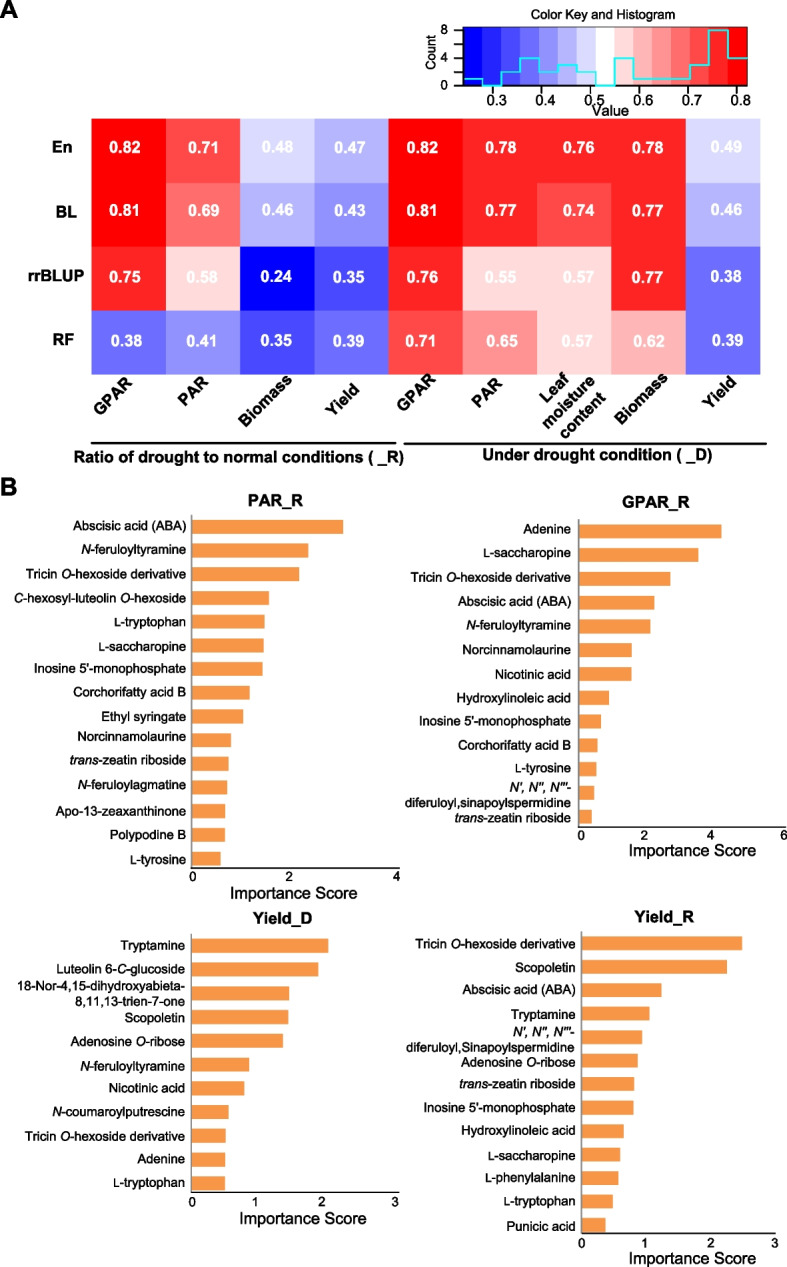
DRMs-based prediction of DR. **A** The performance of DRMs-based prediction of phenotypic traits of evaluating DR capacity, using the four modeling algorithms. The performance was evaluated by the Pearson correlation coefficient (PCC) between the actual phenotypic values and the DRMs-based predicted phenotypic values. **B** The DRMs with the importance scores ranking top 50 in the prediction of PAR_R, GPAR_R, yield_D, and yield_R, respectively. The suffix “_D” “_R” represent the values under drought condition and the ratios (drought / normal), respectively

We performed DRM-based prediction of DR indices using four modeling algorithms including ridge regression Best Linear Unbiased Predictor (rrBLUP), Bayesian-Least Absolute Shrinkage and Selection Operator (BL), Random Forest (RF), and Ensemble (En, an integration of the former three algorithms). Leaf water content, biomass, and yield under drought condition (represented by a suffix “_D”) and their ratio values of drought/normal (represented by a suffix “_R”) were used to evaluate DR capability. Meanwhile, green projected area ratio (GPAR) and perimeter/projected area ratio (PAR), two representative image traits measured by the non-destructive phenotyping facility, were also included in the DR prediction because the two image traits were successfully modeled to evaluate stay-green and leaf-rolling, respectively, at the whole plant level under drought condition, and stay-green and leaf-rolling are closely related to drought tolerance and drought avoidance, respectively (Guo et al. 2018). The DR prediction performance was evaluated using PCC between the predicted and the actual phenotypic values. As expected, the algorithm En outperformed the other three modeling algorithms considering that the En integrates the prediction results of the other algorithms (Fig. 2A and Supplementary Table 5). Therefore, the prediction results of En were focused on in the following sections. The PCC values of DRM-based prediction ranged from 0.46 to 0.82 with a median value 0.76 (Fig. 2A and Supplementary Table 5), suggesting large DR contribution from DRMs. For example, the PCC values of GPAR_R and PAR_R were 0.82 and 0.71, respectively (Fig. 2A). To determine the performance of DRM in predicting DR, we quantified the importance score of each DRM. The DRMs with importance scores ranking top 50 were focused in the following analyses (Supplementary Table 6). Two hormones, ABA and *trans*-zeatin riboside (a bioactive form of cytokinin), performed well in predicting leaf water content_D, PAR_R, GPAR_R, and yield_R (Fig. 2B). We also found that both tryptophan and tryptamine, belonging to tryptamine-metabolites group, well predicted yield_D and yield_R (Fig. 2B). *C*-hexosyl-luteolin *O*-hexoside, a flavonoid, also performed well in the prediction of PAR_R, which is consistent with previous studies showing the role of flavonoids in drought avoidance (Brunetti et al. 2018; Watkins et al. 2017). Besides, the DRMs from other metabolic groups, such as polyphenol, phenolamides, and amino acid also performed well in the DR prediction (Fig. 2B and Supplementary Table 6). These results indicate that the DRMs have promising values in DR prediction and some of them may be further explored as bio-markers for DR.

### Genome-wide association study of DRMs

Since the DRMs showed large contribution to DR and large variations under drought condition, but had little correlations between drought and normal conditions (Supplementary Table 3 and 5), it is of significance to dissect the genetic bases of DRM variations under drought condition. For the 233 DRMs, 84.5% (197/233) metabolites showed high broad-sense heritability (*H*^2^ > 0.5) in the rice population comprised of 510 accessions (Supplementary Table 7), indicating large contributions of genetic components in determining the DRMs’ levels.

**Fig. 3:**
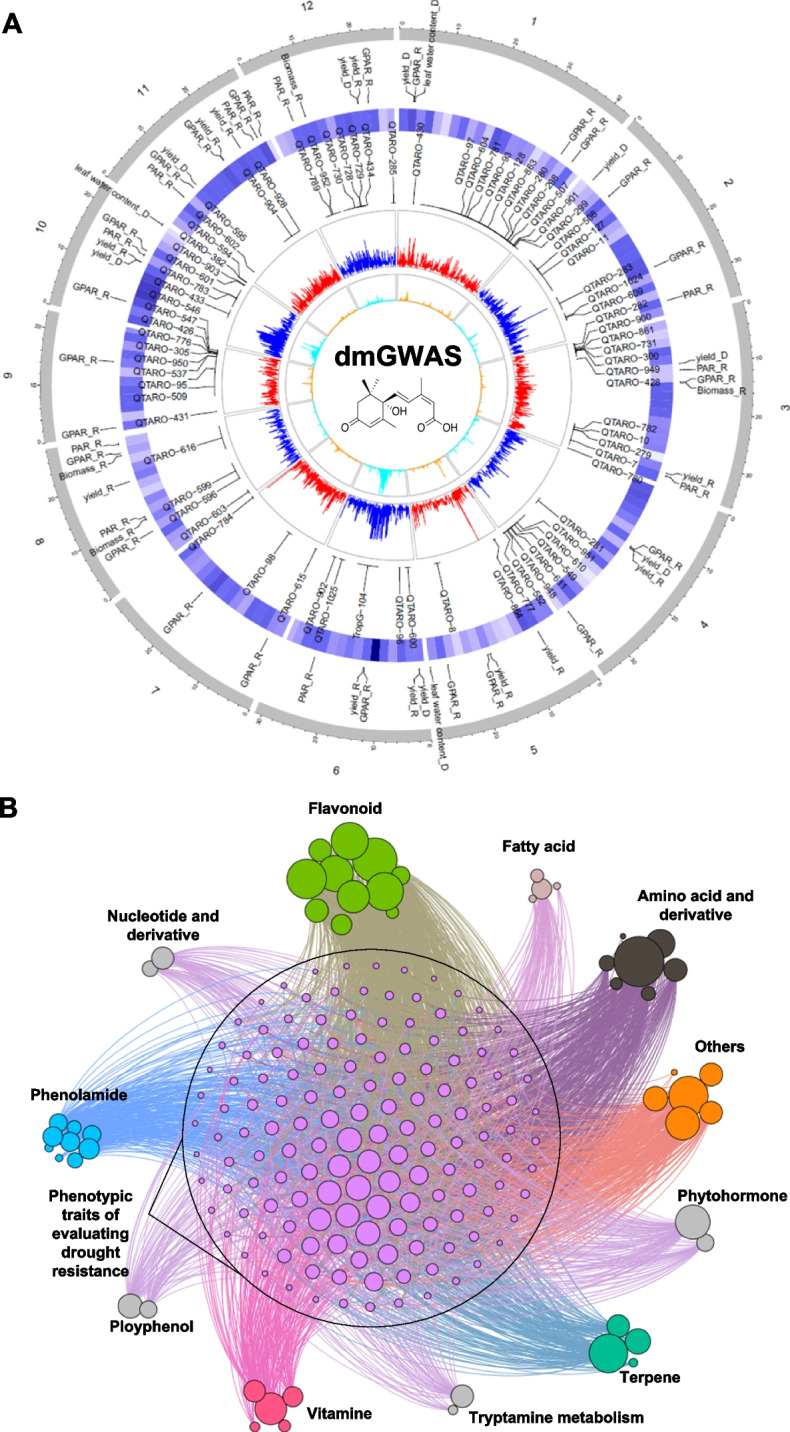
Integration of dmGWAS, dpGWAS, and drQTLs. **A** Circos plot showing dmGWAS loci, co-localized drQTLs from previously reported studies, and co-localized dpGWAS loci of PAR_R and GPAR_R. The five tracks (from inner to outer) represent the number of significant SNPs for each association signal, significance (−log_10_ *P*) of dmGWAS signals, co-localized drQTLs from previously reported studies (partially shown), the number of DRMs associated with each association signal (shown by heat map), and co-localized dpGWAS loci of PAR_R and GPAR_R (partially shown), respectively. **B** Association network of dmGWAS and dpGWAS. In the network, each node represents an annotated DRM or a phenotypic trait of evaluating DR capacity (measured by optical phenotyping facility and manual measurements in our previous study); each edge represents that a DRM and a phenotypic trait are connected by a co-localized locus. Different colors represent different metabolic groups. Each purple circle in the center of the network represents a specific phenotypic trait

To reveal these genetic components, we performed GWAS of 233 DRMs (hereinafter named “dmGWAS” for “drought-responsive metabolites-based GWAS”) using linear mixed model. Based on Bonferroni correction of multiple tests, the genome-wide significance thresholds were set to 1.21 × 10^−6^, 1.66 × 10^−6^, and 3.81 × 10^−6^ in the whole population, *indica* and *japonica* subpopulations, respectively. Considering the linkage disequilibrium (LD) decay distance in rice, the adjacent significant SNPs with high LD to each other (*r*^2^≧0.25) were defined as a locus to avoid the redundancy of association signals. As a result, 2522 significant loci were associated with 233 DRMs in at least one subpopulation (Fig. 3A; Supplementary Table 8). Based on the dmGWAS results, multiple loci controlling a specific DRM or single locus effective for multiple DRMs were found. For example, seven loci distributed on chromosome 1, 2, 6, 7, and 8 were significantly associated with ABA. Among these loci, locus 593 on chromosome 1 was associated with ABA and scopoletin (a DRM belonging to polyphenol group). Ninety-four percent (2382/2522) of dmGWAS loci were co-localized with previously reported DR QTLs (drQTL) in rice (Fig. 3A and Supplementary Table 9), which were retrieved from three public databases (TropGeneDB (Hamelin et al. 2013; Ruiz et al. 2004), QTARO (Yonemaru et al. 2010), and PubMed (https://www.ncbi.nlm.nih.gov/pubmed/)). This result suggests that the causal genes underlying these drQTLs may be related to metabolism. For example, the dmGWAS loci associated with two hormones (ABA and *trans*-zeatin riboside) were co-localized with previous drQTLs controlling leaf water content, leaf rolling score, leaf drying score, relative yield (drought/normal) or yield under drought condition, which is consistent with the results of DRMs-based prediction mentioned above.

Since the population for dmGWAS were simultaneously phenotyped for DR using optical imaging and manual measurement (Guo et al. 2018), the GWAS results of DR-related traits, termed dpGWAS referring to GWAS of the image traits and traditional phenotypic traits under drought condition and the ratio traits of drought/normal, were extracted from previous study and integrated with the dmGWAS of this study to construct a metabolome-phenome association network (Fig. 3B). In the association network, 77% (1939/2522) of dmGWAS loci were co-localized with dpGWAS loci (Supplementary Table 10). For example, the locus 593 (chr1:37480916–37,811,473) was detected by ABA, GPAR_R, and yield_R; and the locus 2155 (chr4:14055749–14,121,776) was detected by tryptamine, yield_R and spikelet fertility_R. These co-localized loci suggest that the physiological indices or yield traits under drought condition may be contributed by these DRMs. We noticed that a DR-related phenotypic trait could be connected with several DRMs in the association network (Fig. 3B and Supplementary Table 10). For example, GPAR and yield_R were connected with 35 and 24 known DRMs, respectively, such as ABA, *trans*-zeatin riboside, and tryptamine. These results suggest that multiple DRMs may collectively contribute to DR-related complex traits.

### Candidate genes of representative dmGWAS loci related to DR

Integrating information of metabolite structure, gene annotation and expression level can accelerate the exploration of candidate genes and facilitate efficient and precise breeding (Chen et al. 2014). Therefore, we further checked whether the integrated analyses could efficiently identify candidate genes for the DRM groups described above, and candidate genes were identified for six representative DRMs as follows.

**Fig. 4:**
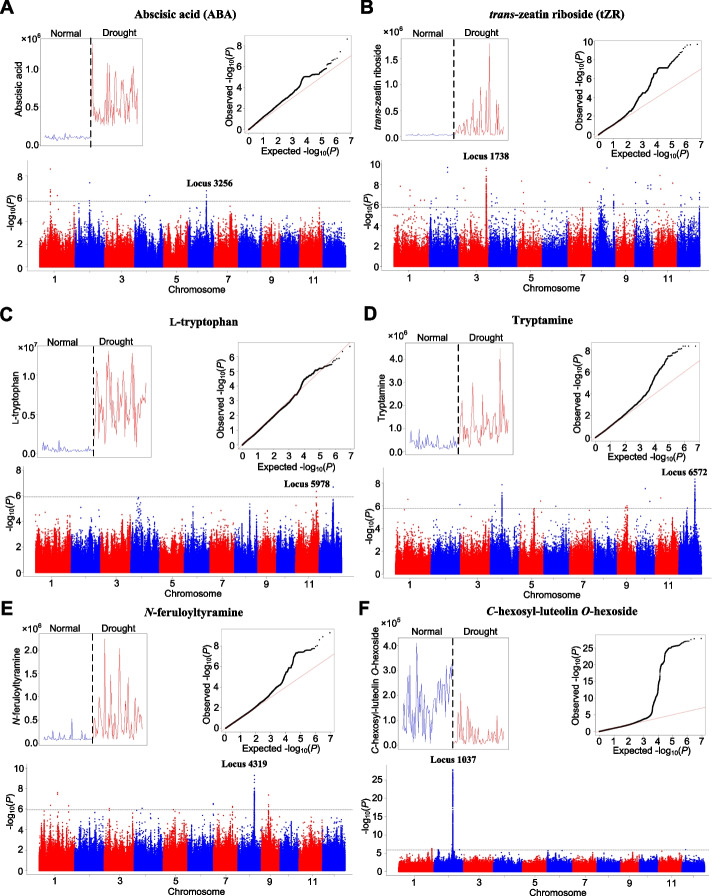
Identification of the candidate genes underlying the metabolic variation of the informative DRMs. The metabolic levels under normal (blue) and drought (red) conditions (the upper left panel), Manhattan plot (the bottom panel) and quantle-quantile plot of dmGWAS (the upper right panel) for abscisic acid (**A**), *trans*-zeatin riboside (**B**), L-tryptophan (**C**), tryptamine (**D**), *N*-feruloyltyramine (**E**), and *C*-hexosyl-luteolin *O*-hexoside (**F)**. For the Manhattan plot, significance values (indicated by -log_10_ *P*) of genome-wide SNPs are plotted against the SNPs’ position on each of 12 chromosomes; the horizontal gray dotted line indicates the genome-wide significance threshold. For the quantile-quantile plot, the observed -log_10_ *P* values are plotted against the expected -log_10_ *P* values; the red line indicates the diagonal

Two known genes related to ABA metabolism were significantly associated with ABA level under drought condition: *LOC_Os07g07050* (*AAO3* (Seo et al. 2000); *P*_LMM_ value: 5.89 × 10^−3^) and *LOC_Os02g47470* (*ABA8ox1* (Krochko et al. 1998); *P*_LMM_ value: 2.27 × 10^−3^). Besides, an association signal for ABA level on chromosome 6 was identified in *indica* sub-population (locus 3256, *P*_LMM_ value: 2.04 × 10^−7^) (Fig. 4A), and this dmGWAS locus co-localized with a previously reported drQTL controlling leaf relative water content under drought condition (Supplementary Table 9) and a dpGWAS locus for yield_R (Supplementary Table 10). In the dmGWAS locus, *LOC_Os06g37150* encodes L-ascorbate oxidase (named “*AO*” (Wu et al. 2017)) and its *cis*-eQTL was identified by GWAS of RNA-seq data under drought condition (*P*_LMM_ = 2.75 × 10^−18^, Supplementary Table 11). The role of *AO* in ABA biosynthesis has been demonstrated by a reported study, which showed that *AO* negatively regulated ABA biosynthesis and that the down-regulation of *AO* enhances salt tolerance in rice (Wang et al. 2021). To explore its function in DR, we generated three knock-out mutant lines of the *AO* gene and found that these mutants showed enhanced DR (Supplementary Fig. 1). This gene may be a causal gene underlying the locus with contribution to the ABA content and drought response.

Cytokinin, another phytohormone, has significant roles in modulating DR (Rivero et al. 2007). In this study, the level of *trans*-zeatin riboside (tZR), a bioactive form of the naturally occurring cytokinin, was elevated with average FC of 7.84 (VIP = 1.22) in the rice population under drought condition. However, the tZR’s level under drought and normal conditions showed no significant correlation (Pearson correlation efficient R = 0.03, *P* > 0.05). The variation of tZR levels under drought condition was much larger than that under normal condition (CV__D_ = 1.52, CV__N_ = 0.34). A dmGWAS locus on chromosome 3 (locus 1738, *P*_LMM_ value: 2.60 × 10^−10^) was identified (Fig. 4B). In the locus, LOC_Os03g58010, annotated as *N*-acetyltransferase, showed down-regulated expression under drought stress (Supplementary Table 12) according to our previous microarray data of two representative rice varieties (IRAT109 and ZS97) at the seedling and reproductive stages (Ding et al. 2013). Further, a strong *cis*-eQTL of the gene was detected under drought condition (*P*_LMM_ = 8.29 × 10^−13^, Supplementary Table 11). These results suggest that *LOC_Os03g58010* may be a candidate gene for transferring acetyl group to tZR and down-regulation of the gene expression under drought condition may elevate tZR level to enhance DR.

In the tryptamine metabolism pathway, tryptophan and tryptamine were representative DRMs that predicted DR very well (Fig. 2B). The metabolic levels of the two DRMs were induced by drought stress (Supplementary Table 3). Similar to most of other DRMs, no significant correlation was detected for metabolic level of tryptophan or tryptamine between drought and normal conditions. For tryptophan, a GWAS locus on chromosome 11 was identified (locus 5978, *P*_LMM_ = 4.62 × 10^−7^) (Fig. 4C). The locus co-localized with a reported QTL controlling leaf rolling score (Supplementary Table 9) and a dpGWAS locus associated with fertile panicle number under drought condition (Supplementary Table 10). In this locus, LOC_Os11g42510 is annotated as tyrosine aminotransferase, which catalyzes the transamination of aromatic amino acids, such as tyrosine and tryptophan (Mehere et al. 2010; Sasidharan and Saudagar 2019). Further, a *cis*-eQTL of the gene was also detected under drought condition (*P*_LMM_ = 3.64 × 10^−7^, Supplementary Table 11). These results indicate that *LOC_Os11g42510* may be a causal gene underlying the tryptophan variation under drought condition.

For tryptamine, two loci on chromosome 12 were identified in the whole population and the *indica* subpopulation, respectively (*P*_LMM_ value: 1.42 × 10^−7^ and 4.50 × 10^−9^ for locus 6344 and 6572, respectively). In the locus 6344, LOC_Os12g16720 was annotated as tryptamine 5-hydroxylase hydroxylating tryptamine (Lu et al. 2022), and it was down-regulated by drought stress (Supplementary Table 12). We speculate that this gene is a possible causal gene and down-regulation of its expression levels might reduce the catabolism of tryptamine and thus enhance DR. In the locus 6572, most genes were annotated as “expressed protein”, “hypothetical protein”, or “retrotransposon protein” (Fig. 4D). Among them, LOC_Os12g32250 was annotated as “WRKY DNA-binding domain containing protein” and was down-regulated at transcriptional level under drought condition (Supplementary Table 12), and this gene might be a causal gene because numerous reported studies have demonstrated the roles of WRKY transcription factors in regulating DR (Jiang et al. 2017).

As a typical DRM from the phenolamide group, *N*-feruloyltyramine could also predict DR. Its metabolic levels were up-regulated upon drought stress (FC = 4.59, VIP = 1.37). GWAS of the DRM identified a strong and clear association signal on chromosome 8 (locus 4319, *P*_LMM_ value: 5.28 × 10^−10^) (Fig. 4E). The dmGWAS locus co-localized with reported drQTLs controlling spikelet sterility under drought condition. In this locus, two linked genes (*LOC_Os08g32160* and *LOC_Os08g32170*) were annotated as 2-oxoglutarate-dependent dioxygenase that convert cinnamate derivatives to coumarins, which is closely related to the chemical structure of *N*-feruloyltyramine (Shimizu 2014). Of the two genes, *LOC_Os08g32160* showed elevated expression levels under drought condition (Supplementary Table 12) and a strong *cis*-eQTL was identified under drought condition (*P*_LMM_ = 7.82 × 10^−12^, Supplementary Table 11). These results indicate that *LOC_Os08g32160* may be a causal gene underlying the increased feruloyltyramine level under drought stress.

*C*-hexosyl-luteolin *O*-hexoside, a DRM among the flavonoid group, showed dramatic reduction at metabolic level under drought condition and it could also predict DR (Supplementary Table 6). A dmGWAS locus with a strong and clear association signal on chromosome 2 was identified (locus 1037, *P*_LMM_ value: 1.56 × 10^−28^) (Fig. 4F), which was also significantly associated with another four DRMs from the flavonoid group (Supplementary Table 8). The locus co-localized with a reported drQTL controlling leaf rolling score under drought condition. In this locus, LOC_Os02g37690, annotated as “UDP-glucoronosyl and UDP-glucosyl transferase”, was reported to be associated with the biosynthesis of flavonoids (Chen et al. 2014). The gene showed down-regulated expression levels upon drought stress (Supplementary Table 12), which is consistent with decrease of the DRM under drought stress. Further, a strong *cis*-eQTL of the gene was discovered under drought condition (*P*_LMM_ = 4.12 × 10^−22^, Supplementary Table 11). Previous studies demonstrate that flavonoid accumulation in guard cells inhibited ABA-induced stomatal closure (Brunetti et al. 2018; Watkins et al. 2017). Therefore, we speculate that *LOC_Os02g37690* may be a causal gene that underlies the decreased flavonoids accumulation and thus promotes the ABA-induced stomatal closure under the drought stress condition.

### Comparative dmGWAS in rice and maize

**Fig. 5:**
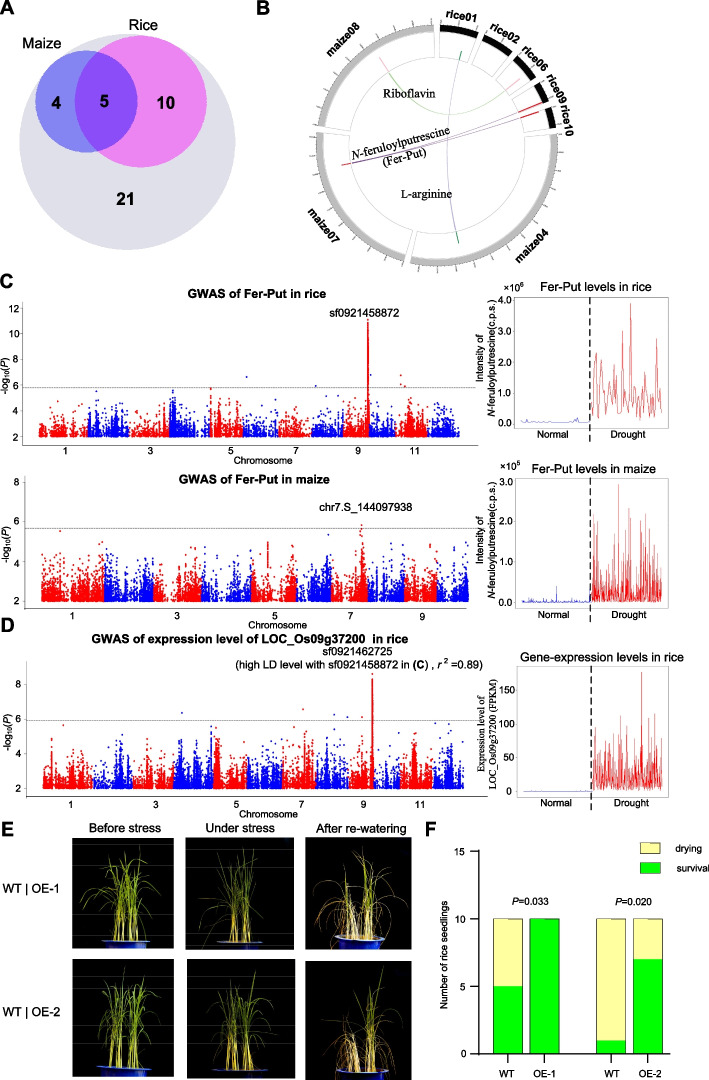
Common DRMs and comparative dmGWAS in rice and maize. **A** Venn plot showing the common DRMs in the two crops based on this rice study and the previously reported study in maize. The grey, purple, and pink circle indicates 40 common metabolites, nine DRMs in maize, 15 DRMs in rice, respectively. **B** Comparative dmGWAS results in rice and maize. The links of Circos plot represent the homologous genes that are significantly associated with the same DRM in rice and maize. Different colors represent different metabolites. The bar’s height in the track of Circos plot represents the significance of association signal (indicate by -log_10_ *P* value). **C** Manhattan plot of dmGWAS of *N*-feruloylputrescine (Fer-Put) in rice (the left upper panel) and in maize (the left bottom panel), the metabolic levels of Fer-Put under normal (blue) and drought (red) condition in rice (the right upper panel) and in maize (the right bottom panel). **D** GWAS of expression levels of *LOC_Os09g37200* under drought condition (the left panel) and its FPKM values based on RNA-seq under normal (blue) and drought (red) conditions (the right panel). **E** Performance of the two independent overexpression lines of *LOC_Os09g37200* under drought stress and after re-watering. **F** Seedling survival rates of the overexpression lines and WT plants after re-watering recovery following drought stress. *P* values are calculated using two-sided fisher’s exact test

Rice and maize are two important representing cereal crops that have been evolved and selected under distinct water regime conditions (lowland- and upland-grown, respectively), but few studies involve the comparison of genetic bases underlying drought responses of the two crops. We wondered whether comparison of the dmGWAS in this study and a reported dmGWAS study in maize (Zhang et al. 2021) can provide metabolic insight into common and specific drought responses of the two crops. Out of 40 common metabolites between the two studies, five common DRMs and 14 specific DRMs (drought-responsive only in one crop) were identified. The common DRMs included two phenolamides (*N*-feruloylputrescine and *N*-feruloyltyramine), two amino acids (L-arginine and L-tryptophan), and one organic acid (caffeoyl shikimic acid) (Fig. 5A), of which the first four DRMs were up-regulated in the two crops under drought condition, but caffeoyl shikimic acid showed an opposite change trend (down- and up-regulated in rice and maize, respectively). Out of the 14 specific DRMs, all the three vitamins were up-regulated in rice (FC = 1.91–4.03) but were not significantly changed in maize (VIP = 0.23–0.88) upon drought treatment. These results suggest that common and specific metabolite-mediated drought responses co-exist in rice and maize. We further compared the dmGWAS results of the common DRMs in the two crops. The comparison revealed four scenarios: two common DRMs (*N*-feruloylputrescine and L-arginine) and their underlying homologous genes showing common drought responses (Fig. 5B); two common DRMs (*N*-feruloyltyramine and L-tryptophan) showing common metabolic responses, but being underlain by non-homologous genes; one specific DRM (riboflavin/vitamin B2) showing drought-responsiveness only in rice, but being underlain by a pair of homologous genes (Fig. 5B); one common DRM (caffeoyl shikimic acid) with opposite metabolic responses and 13 specific DRMs being underlain by non-homologous genes.

As a member of phenolamides group, *N*-feruloylputrescine (Fer-Put for short hereinafter) can predict DR (Supplementary Table 6). Based on the dmGWAS results in the two crops, two genes (*LOC_Os09g37180* and *LOC_Os09g37200*) in the locus 4811 and three genes (*LOC_Os10g01690*, *LOC_Os10g01720*, and *LOC_Os10g01920*) in the locus 4841 in rice are homologs of *GRMZM2G013530* in a locus on chromosome 7 in maize (Fig. 5B-C). All these genes were annotated as hydroxycinnamoyl transferase, which was associated with the chemical structure of Fer-Put (Chen et al. 2014). Interestingly, a strong *cis*-eQTL of LOC_Os09g37200 was detected and it was co-localized with the dmGWAS locus 4811 in rice (Fig. 5D and Supplementary Table 11). The genotype-G group of the gene showed both higher expression levels and higher metabolic levels of Fer-Put than those of the genotype-A group (Supplementary Fig. 2)A; and significant correlation between the gene expression levels and the Fer-Put levels was observed in rice (Supplementary Fig. 2B). These results suggest that the metabolic variation may be attributed to the different transcriptional levels of *LOC_Os09g37200*. In maize, although no significant *cis*-eQTL of *GRMZM2G013530* (a homolog of *LOC_Os09g37200*) was detected (Supplementary Fig. 2)C, the association between the transcriptional levels of *GRMZM2G013530* and the metabolic levels of Fer-Put in maize was consistent with that of *LOC_Os09g37200* in rice (Supplementary Fig. 2)D-F. These results indicate that the common metabolite-mediated drought responses may be underlain by homologous genes.

Although *LOC_Os09g37200* has been confirmed to be responsible for Fer-Put biosynthesis under well-water condition (Chen et al. 2014), its role in drought resistance and the causal variants remains unknown. The overexpression lines of *LOC_Os09g37200* showed enhanced DR based on seedling survival rate after drought stress (Fig. 5E-F). To explore the causal variants, we performed a candidate-gene association analysis and found that the significant variants were enriched in promoter region (Supplementary Fig. 3)A, which was located in the identified *cis*-eQTL (Fig. 5D). Based on the significant variants, two major haplotypes were identified. Among them, two INDELs may disrupt a priori *cis*-elements in the unfavorable haplotype (Supplementary Fig. 3B), which may be the causal variants underlying the metabolic variation of Fer-Put.

Arginine is an important precursor of Fer-Put (Chen et al. 2016), and it could predict stay-green levels of stressed plants (Supplementary Table 6). Based on the comparative dmGWAS of arginine in the two crops, *LOC_Os01g52680* in the locus 500 on chromosome 1 in rice was a homolog of *GRMZM2G446426* in a locus on chromosome 4 in maize (Fig. 5B). Both candidate genes are annotated as MADS transcription factor and belong to the SRF superfamily of MADS-box protein. Based on previous studies, Arg80 (the first MADS-box protein) and Mcm1, belonging to the SRF superfamily, regulate arginine metabolism in *Saccharomyces cerevisiae* (Messenguy and Dubois 2003)*.* We propose that the homologous genes may be responsible for the common arginine-mediated drought responses in rice and maize. Besides, another two common DRMs, *N*-feruloyltyramine and L-tryptophan were up-regulated in rice and maize, but no homologous genes were discovered in the comparative dmGWAS results of the two crops. The results of the common DRMs suggest that the common metabolic responses can be underlain by both homologous and non-homologous genes.

As a specific DRM, riboflavin (vitamin B2), the direct precursor of the cofactors flavin adenine dinucleotide (FAD) and flavin mononucleotide (FMN), could predict stay-green levels of stressed plants (Supplementary Table 6). The metabolite was up-regulated in rice but not responsive in maize under drought condition. Despite this, a candidate gene *LOC_Os06g36400* in a locus on chromosome 6 in rice was a homolog of *GRMZM2G141277* in a locus on chromosome 8 in maize (Fig. 5B). Both genes are annotated as HAD phosphatase, which have been identified and characterized in the riboflavin biosynthesis in *Arabidopsis thaliana* (Sa et al. 2016). Despite divergent metabolic responses in different crops, the metabolic variation of riboflavin under drought condition may be underlain by a pair of homologous genes.

Besides, 13 specific DRMs and one common DRM (caffeoyl shikimic acid) showed divergent drought responses. Different from riboflavin, no homologous candidate genes were identified. Altogether, the comparative dmGWAS results indicate that homologous and non-homologous genes collectively contribute to the common and specific metabolite-mediated drought responses in rice and maize.

## Discussion

DR involves various metabolic and physiological responses to drought stress. It is an extremely complex trait that is controlled by a large number of minor-effect genes with diverse molecular mechanisms, so it is difficult to identify the reliable genetic loci and their causal genes (Hu and Xiong 2014). To solve the dilemma, a rice core collection was phenotyped using high-throughput optics phenotyping facility and genetic mapping was performed in our previous study (Guo et al. 2018). However, diverse molecular mechanisms involved in DR results in insufficient clues/ a priori knowledge for the exploration of causal gene in a locus, which usually spans large confidence interval and thus includes many annotated genes. Previous molecular studies demonstrate that the metabolic responses to drought stress and their underlying genes collectively contribute to DR (Kumar et al. 2021). Therefore, the metabolome could bridge the causal genes and DR as intermediary. In this study, we identified and quantified drought-responsive metabolites, DRMs, in a large rice core collection. Most DRMs could predict DR in high confidence and some DRMs have potentials to be used as bio-markers to select rice materials for DR breeding. Most of dmGWAS loci co-localized with the previously reported drQTLs, suggesting the large contribution of the DRMs to DR. Further, we efficiently identified seven candidate genes for six DRMs, which belong to four metabolic groups, by considering only two factors: i) the logic connection between gene annotation and metabolite structure, and ii) gene expression change pattern under drought condition. Among them, the down-regulation of *AO* gene, which was associated with ABA content variation under drought condition, resulted in enhanced DR (Supplementary Fig. 1). Besides, the comparative dmGWAS in rice and maize facilitated the rapid identification of candidate genes and three pairs of candidate homologous genes underlying the variations of three DRMs were identified. Furthermore, *cis*-eQTLs for seven out of 10 candidate genes were detected by GWAS of RNA-seq data under drought condition (Supplementary Table 11), suggesting that the expression polymorphism at transcriptional level may be a main source of the natural variation of metabolite-mediated drought responses.

Even though rice and maize are originated from a recent common ancestor (Liu et al. 2017; Murat et al. 2017), these two important cereal crops have been evolved under distinct water regime conditions (lowland and upland respectively). Different water conditions could result in different periods of water deficiency, which may promote the two crops to evolve divergent mechanisms to resist short- and long-term drought stress. Besides, rice and maize belong to C3 and C4 plants, respectively, which divergently respond to drought stress in stomatal development (Song et al. 2023). During domestication and genetic improvement, the lowland rice undergoes directional selection for high yield potential during domestication since it is mostly grown in the field with sufficient water, sometimes with irrigation equipment, and thus faces low risk of drought stress (Xia et al. 2019). By contrast, the maize grown in drought-prone field may undergo bi-directional selection for high yield potential and DR. Due to trade-offs between DR and yield potential, bi-directional selection may result in balancing selection in maize. Divergent selection patterns of rice and maize correspondingly result in divergent drought responses. However, few studies eyed on the comparison of drought responses between rice and maize. In this study, for the first time, we integrated the DRM GWAS in rice with the similar study in maize (Zhang et al. 2021) to find the common and distinct metabolic responses between rice and maize. As a proof, Fer-Put levels were up-regulated under drought condition in the two crops and the metabolic variations were underlain by expression differences of a pair of homologous genes encoding hydroxycinnamoyl transferase. And the gene was demonstrated to enhance DR in rice (Fig. 5E-F), which may enhance DR in other crops. The comparison of metabolite-mediated drought responses is a promising approach to explore crucial DR-related genes and to enhance DR of multiple crops efficiently.

In future, unknown DRMs need to be identified to reveal a more complete landscape of drought response. As an alternative approach, integration of gaussian graphical modeling (GGM) network and mGWAS may facilitate the annotation of unknown metabolites (Chen et al. 2016). We tried to construct a GGM network and to integrate it with the dmGWAS results. As a proof, an unknown DRM, gl0238 (FC = 2.11, VIP = 1.88) was successfully annotated. This DRM was connected with *trans*-zeatin *N*-glucoside (*q* = 4.22 × 10^−7^) in the GGM network, and the dmGWAS loci of gl0238 and *trans*-zeatin *N*-glucoside were co-localized. In this locus, *LOC_Os05g12450* was annotated as “UDP-glucosyltransferase” and showed expression response to drought stress (Supplementary Table 12). A *cis*-eQTL locus of *LOC_Os05g12450* was co-localized with the dmGWAS locus (Supplementary Table 11). It is speculated that *LOC_Os05g12450* is a causal gene and its encoded UDP-glucosyltransferase converts gl0612 to *trans*-zeatin *N*-glucoside and thus gl0612 could be *trans*-zeatin, a bioactive form of the naturally occurring cytokinins. The example highlights the potential of integrating GGM and dmGWAS for the identification of unknown DRMs, though further confirmation is needed.

## Methods

### Plant materials and experimental design

A total of 510 rice (*Oryza Sativa*) accessions comprised of landraces and elite varieties were used in the study. The population was re-sequenced at the genome level using an Illumina Hiseq 2000 platform to obtain genotypic information, which can be retrieved and downloaded from the RiceVarMap database (http://ricevarmap.ncpgr.cn/v1), of which the reference genome is Nipponbare (*Oryza sativa* L. ssp. *japonica*) of MSU Rice Genome Annotation Project Release 6.1. To avoid the bias of DR evaluation caused by heading date variation, the rice accessions were assigned into two groups based on the heading date, and the germination dates were staggered to ensure flowering synchrony for the two groups. Clean seeds were soaked in water for 1 d and then incubated for 1 d. The pre-germinated seeds were sown in the field and 20-day-old seedlings were transplanted to the greenhouse of a high-throughput rice phenotyping facility at Huazhong Agricultural University, Wuhan, Hubei Province, China (30°28′N 114°20′E). Four healthy plants for each accession were grown in four pots filled with 4.5 kg of soil per pot, which was air-dried, pulverized, and well-mixed with organic fertilizer in advance. After transplanting, irrigation was performed to keep standing water of 3-5 cm depth in pots. Another two plants of each accession were needed to trace panicle development in a destructive way. When a rice accession grew to the booting stage (panicle elongation), the irrigation was stopped to start drought stress for the accession. The soil water content was dynamically monitored by TRIME-PICO32 (IMKO Micromodultechnik GmbH, Ettlingen, Germany) on the basis of time domain reflectometry (TDR). When the soil water content decreased to 15% (TDR value), the plants were watered once per day to keep the soil water content at 15% (TDR value) for 5 days. Then the plants were phenotyped using an optics-based high-throughput phenotyping facility (reported in our previous study), followed by leaf sampling for metabolite profiling. For the four plants of each accession, the first upper mature leaves of each two plants were harvested and combined into one tube (frozen in liquid nitrogen in advance) as one biological replicate and thus there were two biological replications for the following metabolite profiling. To identify the drought-responsive metabolites (DRMs), four plants of 60 extremely drought-responsive rice accessions (including 30 *indica* and 30 *japonica* accessions) from the rice core collection, were grown under normal (non-stress) condition and their leaf samples were collected as those under drought condition.

### Metabolite profiling

The freeze-dried leaves were crushed using a mixer mill (MM 400, Retsch) with a zirconia bead for 1 min at 30 Hz. And 100 mg dried powder of each rice accession was weighed and extracted overnight at 4 °C with 1.0 ml pure methanol (or 70% aqueous methanol) containing 0.1 mg L^−1^ lidocaine for lipid- and water-soluble metabolites, followed by analyses of LC-MS. A scheduled multiple reaction monitoring (sMRM) method of AB SCIEX QTRAP 5000 LC-MS system was used for the quantification of metabolites. The detailed procedures of sample preparation, metabolites extraction, construction of the MS^2^ spectral tag (MS2T) library, metabolites annotation and quantification, have been described in the previous study (Chen et al. 2013).

### Statistical analyses

To show the effect of drought stress on rice metabolome, principle component analysis (PCA) was performed using R package “factoextra” and “FactoMineR” and linear discriminant analysis was performed using R package “MASS”. To identify DRMs, the variable importance for the projection (VIP) of OPLS-DA (using R package “ropls”) and false discovery rate (FDR) of paired *t*-test between the drought and normal conditions were calculated; the metabolite of VIP > 1 and FDR < 0.001 was defined as DRM. The enrichment scores of DRMs were calculated and plotted using a customized R script, which is available on request. The metabolome-based prediction of DR capacity was performed using four modeling algorithms including ridge regression Best Linear Unbiased Predictor (rrBLUP), Bayesian-Least Absolute Shrinkage and Selection Operator (BL), Random Forest (RF), and Ensemble (En, an integration of the former three algorithms). The prediction performance was evaluated by the Pearson correlation coefficient between the predicted phenotypic values and the actual values. The influence of a metabolite on the prediction performance was evaluated by the importance scores and the DRM with the importance scores ranking top 50 was considered as informative DRM. The scripts for the four modeling algorithms were from the previous study (Azodi et al. 2020). Paired-sample or independent-sample *t*-test, Kruskal-Wallis one-way ANOVA, and Pearson correlation coefficients (R) were performed or calculated using IBM’s SPSS version 19 (IBM Corp., Armonk, USA). Fisher’s exact test was performed using R program.

### Genetic analyses

The broad-sense heritability (*H*^2^) was calculated using ANOVA (one-way analysis of variance) based on the phenotypic data: *H*^2^ = V_G_ / (V_G_ + Ve / N); phenotypic variance was partitioned into genotype (V_G_) and environment (Ve); N represents the number of biological replications (*N* = 2 in this study). The best linear unbiased prediction (BLUP) values for each metabolite were calculated for genome-wide association study (GWAS). SNPs of the minor allele frequency (MAF) less than 0.05 were removed and a total of 4,358,600, 2,863,169, and 1,959,460 SNPs remained for GWAS in the whole, *indica*, and *japonica* populations, respectively (Guo et al. 2018). In GWAS, a linear mixed model was adopted using the factored spectrally transformed linear mixed models software (FsST-LMM) (Lippert et al. 2011). The genome-wide threshold was calculated as 1/Ne (Li et al. 2013), of which Ne is the effective number of SNPs calculated by GEC software (Li et al. 2012). The Ne is 829,451, 602,309, 262,222 for the whole population, *indica* and *japonica* subpopulations, respectively. As a result, the thresholds were set to 1.21 × 10^−6^, 1.66 × 10^−6^, 3.81 × 10^−6^ in the whole, *indica*, and *japonica* populations, respectively. The independent lead SNPs were determined by removing LD-causing redundant SNPs using “--clump” function of Plink (Purcell et al. 2007). The confidence interval (genomic region) of an association signal was determined using “--clump-r2” “--clump-kb” “--clump-range” functions of Plink (Purcell et al. 2007). The loci with overlapped confidence internals were considered as co-localized loci. A Circos plot was drawn using an R application “shinyCircos” (Yu et al. 2018). A “locus-trait” association network was constructed using Gephi (https://gephi.org/). Gene synteny and collinearity between rice and maize were performed using MCScanX (Wang et al. 2012). Previously reported DR-related QTLs were retrieved from TropGeneDB (http://TropGeneDBdb.cirad.fr/TropGeneDB/JSP/interface.jsp?module=RICE), QTARO (http://qtaro.abr.affrc.go.jp/), and PubMed (https://www.ncbi.nlm.nih.gov/pubmed/). Gaussian graphical modeling (GGM) was performed using R package “GeneNet” and “DMwR” as the previous study (Krumsiek et al. 2012).

### Expression levels of candidate genes

The expression data under drought and normal conditions for two rice varieties (IRAT109 and ZS97) were collected based on Affymetrix GeneChip from a previous study (Ding et al. 2013). The Fragments Per Kilobase Million (FPKM) values were calculated from RNA-seq data using standard protocol of Hisat2 and Stringtie (Pertea et al. 2016).

### Function confirmation of candidate genes

To confirm the role of *LOC_Os09g37200* in drought resistance in rice, overexpression lines were generated by directionally inserting the full cDNAs from Nipponbare first into the entry vector pDONR207 and then into the destination vector pJC034 using the Gateway recombination reaction (Invitrogen) (Supplementary Table 13). The sequence-confirmed constructs were transformed into *Agrobacterium tumefaciens* strain EHA105 and then transferred into the rice variety Zhonghua 11 (Chen et al. 2014; Hiei et al. 1994). The expression levels of *LOC_Os09g37200* in transgenic lines were quantified using primers *AT*-exp-F (5′-CTTCATGCCGTCCTACTTCC-3′) and *AT*-exp-R (5′-GAGGTTGTGGTCGAAGA CG-3′) (Supplementary Fig. 4). Overexpression lines of *LOC_Os09g37200* and wild-type plants (WT) were planted in pots and treated with drought stress at the 4-leaf stage. The stressed seedlings were re-watered and the survival rates were calculated. The *P* values were calculated using two-sided fisher’s exact test.

To confirm the role of *LOC_Os06g37150* in drought resistance in rice, three independent CRISPR lines were developed. A CRISPR vector was constructed using a tandemly arrayed tRNA-gRNA editing system (Xie et al. 2015) using primers UGW-U3-F (5′-GACCATGATTACGCCAAGCTTAAGGAATCTTTAAACATACG-3′) and UGW-gRNA-R (5′-GGACCTGCAGGCATGCACGCGCTAAAAACGGACTAGC-3′). The sequence-confirmed construct was transformed into Zhonghua 11 via *Agrobacterium*-mediated transformation. We examined the genotypes of transformed materials using primers *AO*-seq-F:5′-TGGGCGGACGGGACGGCAT-3; *AO*-seq-R:5′-TCTTACCCCTTGAATCTTGACG-3′. CRISPR lines of *LOC_Os06g37150* were planted in pots. When rice plants grew to the panicle elongation stage (the most sensitive stage to drought stress) and the soil water content decreased to 15% (the TDR value was measured by using a TRIME-PICO32 (IMKO Micromodultechnik, Ettlingen, Germany)), the CRISPR lines and WT plants were phenotyped by our optics-based phenotyping platform before drought stress treatment. Irrigation was then stopped to impose drought stress. Under drought stress condition, the plants were phenotyped again by the phenotyping platform and the image traits were extracted.

### Supplementary Information

**Additional file 1:** **Supplementary Fig. 1.** Functional validation of AO (LOC_Os06g37150) in drought resistance in rice. **Supplementary Fig. 2.** Contribution of transcriptional level polymorphisms of LOC_Os09g37200 and GRMZM2G013530 to the metabolic variation of Fer-Put in rice and maize, respectively. **Supplementary Fig. 3.** Possible causal variants of LOC_Os09g37200 underlying the Fer-Put variation. **Supplementary Fig. 4.** Relative expression levels of LOC_Os09g37200 in the overexpression lines and WT plants.

**Additional file 2:** **Supplementary Table 1.** Information of 682 metabolic features identified in the study. **Supplementary Table 2.** Metabolic quantification data of 60 rice accessions under drought and normal conditions. **Supplementary Table 3.** Statistical analyses regarding drought responses of metabolites. **Supplementary Table 4.** Metabolic quantification data of 510 rice accessions under drought condition. **Supplementary Table 5.** Performance of DRMs-based prediction of drought-resistance evaluating index using four modeling algorithms. **Supplementary Table 6.** Rank of prediction performance of individual DRM. **Supplementary Table 7.** Coefficient of variance and broad-sense heritability of individual DRM in a large rice population containing 510 accessions. **Supplementary Table 8.** GWAS of drought-responsive metabolites using linear mixed model (LMM). **Supplementary Table 9.** dmGWAS (DRMs-associated GWAS) loci overlapped with drQTLs (previously reported QTLs of drought resistance). **Supplementary Table 10.** dmGWAS (GWAS of DRMs) loci overlapped with dpGWAS (GWAS of DR-related phenomic data) loci. **Supplementary Table 11.** Cis-eQTLs identified by GWAS of RNA-seq data of candidate genes under drought condition. **Supplementary Table 12.** Fold change of gene expression levels of drought/normal based on our previous microarray data. **Supplementary Table 13.** Primers used in this study.

## Data Availability

All data and materials are available in the paper and online supplementary files.
