# Supplementary material for: Common and specific genetic basis of metabolite-mediated drought responses in rice
Source: Stress Biol. 2024 Jan 23;4(1):6. doi: 10.1007/s44154-024-00150-4 (PMC10803723; doi:10.1007/s44154-024-00150-4)
Supplement: Supplementary file 1 — Additional file 1: Supplementary Fig. 1. Functional validation of AO (LOC_Os06g37150) in drought resistance in rice. Supplementary Fig. 2. Contribution of transcriptional level polymorphisms of LOC_Os09g37200 and GRMZM2G013530 to the metabolic variation of Fer-Put in rice and maize, respectively. Supplementary Fig. 3. Possible causal variants of LOC_Os09g37200 underlying the Fer-Put variation. Supplementary Fig. 4. Relative expression levels of LOC_Os09g37200 in the overexpression lines and WT plants. [file 44154_2024_150_MOESM1_ESM.docx]

**SUPPLEMENTARY FIGURES**


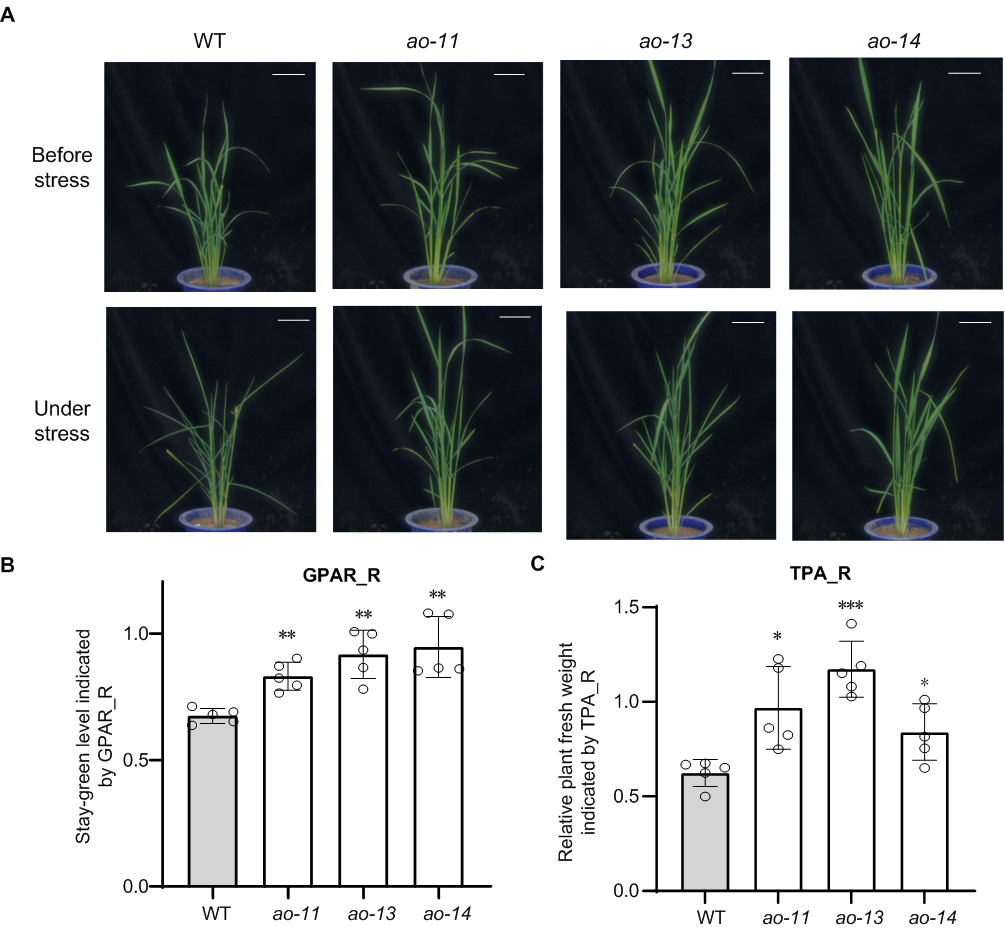


**Supplementary Fig. 1** Functional validation of *AO* (LOC_Os06g37150) in drought resistance in rice.

1. Performance of three CRISPR lines of *AO* and WT plants at the reproductive stage before and under drought stress condition. The images were acquired by the optics-based phenotyping platform. Scale bars, 0.10 m.
2. Stay-green levels indicated by the image trait GPAR_R (green projected area ratio of plant _R) of the CRISPR lines of *AO* and WT plants.
3. Relative plant fresh weight indicated by the image trait TPA_R (total projected area of plant _R) of the CRISPR lines of *AO* and WT plants.

The label “_R” represents the ratio of image trait under / before drought stress condition. Data is presented as mean value ± SD and asterisks indicate significance levels (****P* < 0.001, **0.001 ≦*P* < 0.01, *0.01 ≦*P* < 0.05; two-sided *t*-test).

**
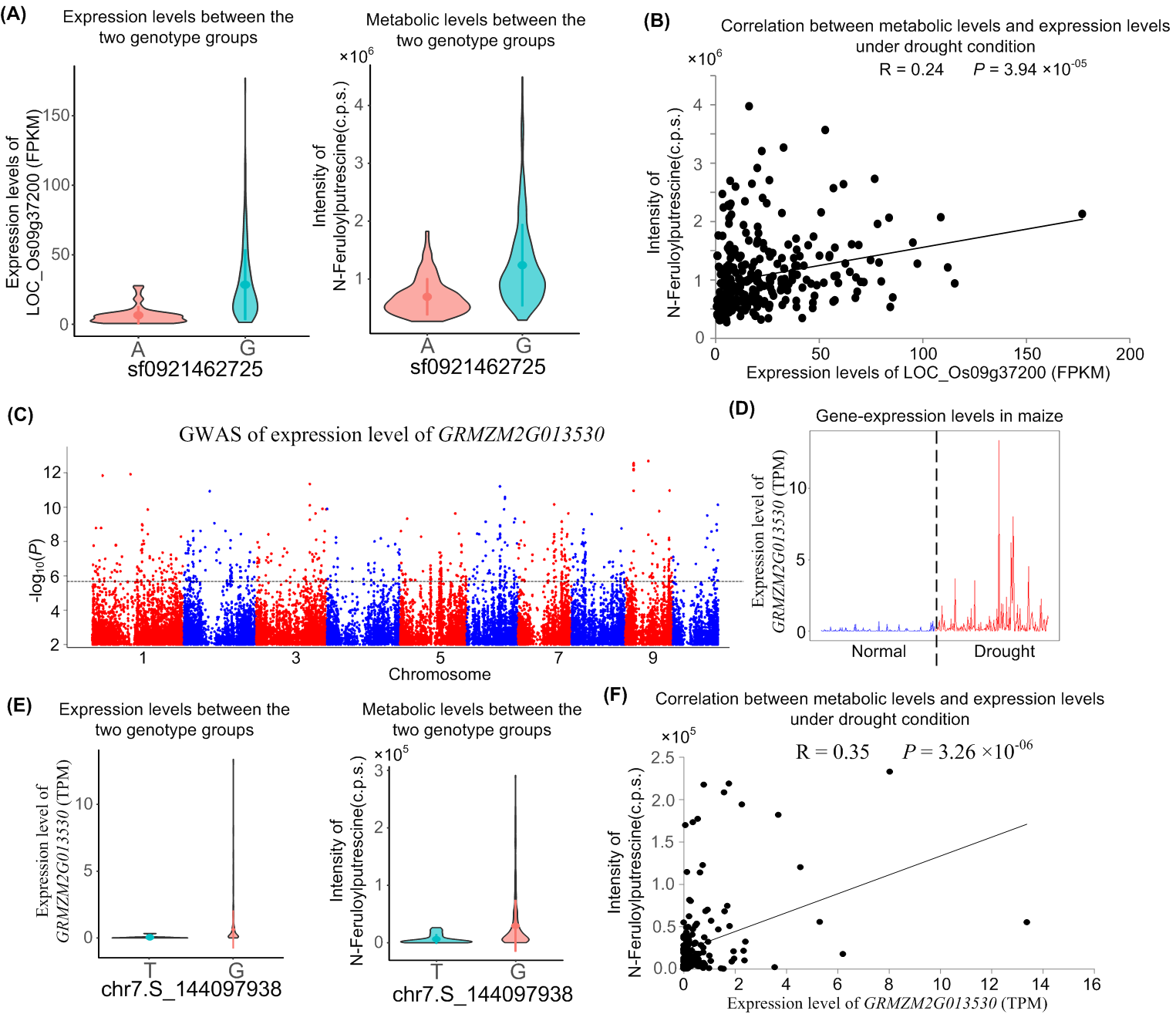
**

**Supplementary Fig. 2** Contribution of transcriptional level polymorphisms of *LOC_Os09g37200* and *GRMZM2G013530* to the metabolic variation of Fer-Put in rice and maize, respectively.

1. Expression levels (FPKM values) of *LOC_Os09g37200* and metabolic levels of Fer-Put between the two genotype-groups of the lead SNP sf0921462725. The *P* values were calculated using two-sided *t*-test.
2. The correlation between the expression levels of *LOC_Os09g37200* and metabolic levels of Fer-Put.
3. Manhattan plot of eGWAS of *GRMZM2G013530* under drought condition in maize.
4. Gene expression levels revealed by RNA-seq data at population scale under normal and drought conditions.
5. Expression levels (TPM values) of *GRMZM2G013530* and metabolic levels of Fer-Put between the two genotype-groups of the lead SNP chr7.S_144097938. The *P* values were calculated using two-sided *t*-test.
6. The correlation between the expression levels of *GRMZM2G013530* and metabolic levels of Fer-Put under drought condition in maize.


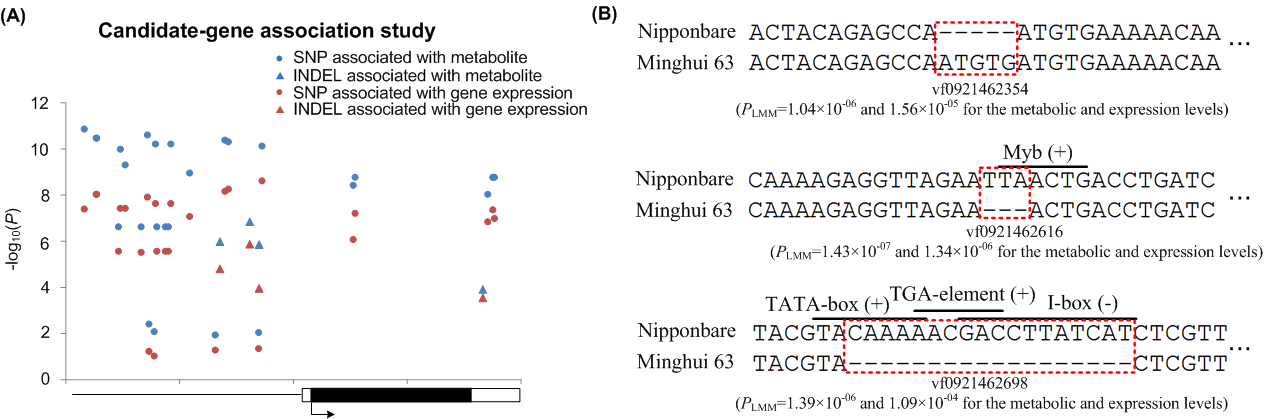


**Supplementary Fig. 3** Possible causal variants of *LOC_Os09g37200* underlying the Fer-Put variation.

1. Candidate-gene association study of *LOC_Os09g37200* and Fer-Put levels under drought condition.
2. Promoter sequence alignment of two haplotypes of *LOC_Os09g37200*. Both Nipponbare and Minghui 63 have reference-grade genome sequence, of which the former corresponds to the favorable haplotype (corresponding to higher levels of Fer-Put) and the latter corresponds to the unfavorable haplotype.


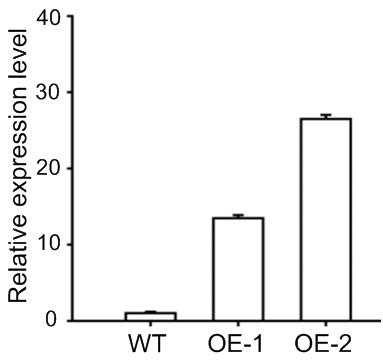


**Supplementary Fig. 4** Relative expression levels of *LOC_Os09g37200* in the overexpression lines and WT plants.

Data is presented as mean value ± SEM (n=3).
